# Food activities and identity maintenance in old age: a systematic review and meta-synthesis

**DOI:** 10.1080/13607863.2014.971707

**Published:** 2014-11-06

**Authors:** Nicola Ann Plastow, Anita Atwal, Mary Gilhooly

**Affiliations:** ^a^ Division of Occupational Therapy, Stellenbosch University, Cape Town, South Africa; ^b^ Division of Occupational Therapy, Centre for Professional Practice Research (CPPR), Brunel University, London, UK; ^c^ Brunel Institute for Ageing Studies (BIAS), Brunel University, London, UK

**Keywords:** identity, maintenance, food, nutrition, active ageing

## Abstract

**Objectives:** Services provided to older people should be developed based on active ageing policies. Nutrition is one aspect of active ageing, but little is known about how food activities contribute to psychological well-being in later life. This is a systematic review of qualitative and quantitative research that answers the question ‘What is known about the relationship between food activities and the maintenance of identities in old age?’.

**Methods:** We followed the preferred reporting items for systematic reviews and meta-analyses guidelines and used quality assessment parameters to complete a systematic review and narrative synthesis. Academic Search Premier, MEDLINE, CINAHL Plus, and PsycINFO databases were searched.

**Results:** We initially identified 8016 articles, of which 167 full-text articles were screened for inclusion. Twenty-two articles were included in the review. There was moderate evidence from nine qualitative and two quantitative studies, of variable quality, that food activities contribute to the maintenance of women's gendered identities, the ethnic identities of men and women, and community identities. There was moderate evidence from 10 qualitative studies, of variable quality, that a change in food choice and deteriorating health changed food activity participation. These changes threatened identities. Most studies included both younger adults and older adults.

**Conclusion:** In later life, there are many life experiences leading to change. Further research is needed to develop understanding of how identity and mental well-being are maintained, despite changes in everyday activities like cooking and eating. This may enable health care professionals to meet psychological needs alongside biological needs during nutritional interventions.

## Introduction

Active ageing is currently ingrained within health and social care policy in relation to best practice for older adults in the United Kingdom. The active ageing policy framework of the World Health Organization (World Health Organization, 2002) suggests that active ageing includes more than reducing disease and disability. Instead, active ageing is a ‘process of optimising opportunities for health, participation, and security in order to enhance quality of life as people age’ (World Health Organization, 2002). The terms ‘successful ageing’ and ‘living well in later life’ are preferred by Wistow, Waddington, and Godfrey (2003), who felt these terms better reflected the concept by relating to more than just physical aspects of well-being. Participating in meaningful and satisfying activities contributes to quality of life across all three major theories of active ageing (World Health Organization, 2002), successful ageing (Rowe & Kahn, 1997), and productive ageing (Butler & Gleason, 1985).

Many meaningful and satisfying activities involve food. We defined food activities as any task, action, or life experience involving food. These can include acquiring food, eating, drinking, preparing meals, and managing diet (World Health Organization, 2001). Food activities are personally meaningful because they play a symbolic role in the way adults view themselves as individuals (Pietrykowski, 2004), as part of their families (Fiese et al., 2002), and as members of society (Devine, Sobal, Bisogni, & Connors, 1999; D’Sylva & Beagan, 2011; Locher et al., 2005). Engaging in meaningful activities is important for maintaining autonomy and a sense of personal identity for older people (Phinney, Chaudhury, & O’Connor, 2007). At the same time, there are many changes in later life that may limit a person's ability to do their food activities, restrict their participation in situations involving food, and increase the risk of malnutrition. Despite the multiple meanings of food activities, a biomedical approach to food and nutrition in later life is evident across the literature.

As meaningful activities, food activities may contribute in a positive way to an individual's experience of their important identities. Identity is a multidimensional construct that is understood in a variety of ways across the literature (Vignoles, Schwartz, & Luyckx, 2011). In our study, we initially drew on Christiansen's (1999, p. 577) theoretical paper in which he defines of identity as ‘the person we think we are’. This includes feelings and ideas about ourselves (the self), conclusions or inferences about ourselves (self-concept), and how we evaluate ourselves (self-esteem), within the context of our interpersonal relationships and daily lives (Christiansen, 1999). Identity also includes thoughts about who we were in the past, who we are in the present, and who we might become in the future (Christiansen, 1999; Markus & Nurius, 1986; Vignoles et al., 2011). In some parts of the literature, the term ‘identity’ refers to specific characteristics of a person, such as ‘ethnic identity’ or ‘gender identity’. In other parts of the literature, both the terms ‘identity’ and ‘self’ refer to a composite sense of who one is (Christiansen, 1999; Whitbourne & Collins, 1998). In keeping with the use of the term ‘identity’ in many of the papers in this review, we use the term ‘identity’ to refer to any of the many components that contribute to ‘the person we think we are’. The term ‘composite sense of self’ is used to refer to an overall sense of who one is.

Christiansen (1999) argues that participation in important and meaningful activities contributes to the maintenance of a composite sense of self in later life. He also emphasises the importance of a consistent life story or narrative. Identity maintenance is a person's ability to preserve a consistent and stable view of who they are across the past, the present, and the future. Some literature refers to the maintenance of component identities, while other refers to maintenance of a composite sense of self. Identity maintenance is important for mental well-being in later life because it is associated with higher self-esteem, and more positive experiences of ageing (Westerhof, Whitbourne, & Freeman, 2012), and increased longevity (Levy, Slade, Kunkel, & Kasl, 2002). Christiansen (1999) also suggests that ageing, disability, and ill-health contribute to a loss of meaning in daily activities. This loss of meaning subsequently leads to a change in self and identities. Changes in identity have the potential to reduce mental well-being because changes in identity are associated with an increase in neurotic self-reflection and low self-esteem (Sneed & Whitbourne, 2003), and explain a part of the relationship between physical symptoms and depression in later life (Weinberger & Whitbourne, 2010). Significantly, the same life experiences that lead to changes in food activities may also threaten and change identities.

Although both food activities and identity maintenance contribute to active ageing and improved quality of life, little is known about how food activities may contribute to either maintenance or change in identities in later life. This is an important gap in the literature because food activities are such an essential part of daily life. The aims of this systematic review were to (1) describe existing knowledge, (2) evaluate the strength of evidence, and (3) identify gaps in existing research on the relationship between food activities and identity maintenance and change in later life.

## Methods

We conducted a mixed-methods systematic review to meet our aim. A mixed-methods systematic review includes both qualitative and quantitative studies. Harden (2010) argues that this type of review is more useful, has greater impact, and is more likely to maximise the findings of a review.

### Search strategy

The PRISMA (preferred reporting items for systematic reviews and meta-analyses) guidelines for systematic reviews were followed. This is an evidence-based minimum set of 27 items for the reporting of systematic reviews. The PRISMA guidelines include four phases of identification, screening, assessment of eligibility, and inclusion (Moher, Liberati, Tetzlaff, & Altman, 2009).

The key questions guiding the review were:

1. What is the relationship between food activities and the maintenance of identities in later life?
2. What is the relationship between food activities and a change in identities in later life?

We used the research databases Academic Search Premier, CINAHL Plus, MEDLINE, and PsycINFO. Papers published up to March 2014 were obtained. The keywords used in combination were self and identity, identity, identities, possible selves, and food. The keywords self and identity, identities, and possible selves were used to capture the past, present, and future aspects of identity from a range of theoretical perspectives. Limits were placed on this search. These limits were keywords in abstract, aged over 65 years, peer reviewed journal, English language, and human participants. Secondary searches were carried out using the keywords for identity (identity, possible selves) and keywords for food activities. The food activity keywords used were grocery, cook, meal preparation, eat, nutrition, and grow. Reference lists of the review papers were also scanned for additional papers.

We screened the study titles and abstracts of the limited and secondary searches to determine which articles met the following inclusion criteria:

1. included adults over age 65,
2. any aspect of food activity included in study design or findings,
3. for quantitative studies: a measure of identity included in study design,
4. for qualitative studies: themes related to identity evident in study findings.

Exclusion criteria were:

1. studies including only older adults living in nursing or residential care,
2. studies that did not investigate the concept of identity, but made general statements about food identities in the discussion or conclusion,
3. studies including animal subjects or focused on nutritional properties at a biochemical level,
4. duplicate studies,
5. grey literature.

No studies were excluded on the basis of the methods that were used. The full text of the articles that met the inclusion criteria was reviewed in more depth to determine if the inclusion and exclusion criteria were met.

### Synthesis of findings

We took a meta-ethnographic approach to the synthesis of the findings. Within this approach, the aim was not to aggregate the findings, but rather to reach a new interpretation of the relationship between food activities and the maintenance of identities (Harden, 2010).

For the qualitative studies, we used the narrative synthesis methods of Arai et al. (2007), in which words and text were used to summarise and explain the findings. First, we used textual description to systematically summarise included studies. Next, key characteristics of the studies were tabulated to begin comparison and identification of patterns between the studies. Then, the studies were grouped according to overarching identity themes. Thematic analysis was conducted within these overarching themes to identify salient or recurrent themes, by reading and re-reading the findings or results section of each article.

A meta-analysis was planned for the quantitative studies. Too few papers of sufficient methodological rigour were identified. The narrative findings of the quantitative papers were incorporated within the narrative synthesis of the qualitative studies.

### Quality assessment

The robustness of evidence supporting a relationship between food activities and maintenance of identities was then assessed in two ways. First, the quality of the articles selected for review was assessed using the six quality assessment parameters for the qualitative studies, and six assessment parameters for the quantitative studies, described by Annear, Keeling, Wilkonson, Gidlow, and Hopkins (2014) (see Tables 1 and 2). A score of 3 was allocated in each parameter, where the study met high standards of rigour. A score of 0 was allocated if the parameter was not described or poorly described or justified in each study. Annear et al. (2014) consider a score <9 to be methodologically weak. Second, the strength of evidence was assessed based on the number of studies published, the overall quality of the studies, the context in which the studies had been conducted, and the consistency of the findings between the studies.

**Table 1.** Quantitative assessment parameters (Annear et al., 2014, p. 596).

	Assessment score			
Assessment parameters	0	1	2	3
Research design	NR/IN	Cross-sectional/quasi-experimental design	Longitudinal	Randomised controlled trial
Reliability and validity of measures	NR/IN	Reliability and validity of some measures ascertained	NA	Pilot testing/prior verification of all measures
Sample size and representativeness	NR/IN	Small sample size	Sample size > 500 (power requirements not reported)	Representative sample (power requirements reported)
Response rate	NR/IN	<60%	60%–79%	>80%
Appropriateness of statistical analysis	NR/IN	Generally appropriate but some inconsistencies	NA	All hypotheses and objectives adequately addressed
Control of potential confounders	NR/IN	NA	NA	Potential confounders included in the analysis

Notes: NR: not reported. IN: inappropriate in the context of the study.

**Table 2.** Qualitative assessment parameters (Annear et al., 2014, p. 596).

	Assessment score			
Assessment parameters	0	1	2	3
Research design	NR/IN	NA	NA	Appropriate to the aims of the study
Sampling and recruitment strategy	NR/IN	NA	NA	Appropriate to the aims of the study
Theoretical framework use	NR/IN	NA	NA	Theoretical framework for methods or design present
Evidence of reflexivity	NR/IN	NA	NA	Preconceptions or meta-positions are addressed
Rigour of data analysis	NR/IN	NA	NA	Well-documented and systematic process
Validation of findings	NR/IN	NA	NA	Triangulation and verification of results

Notes: NR: not reported. IN: inappropriate in the context of the study.

## Results

**Figure 1.**
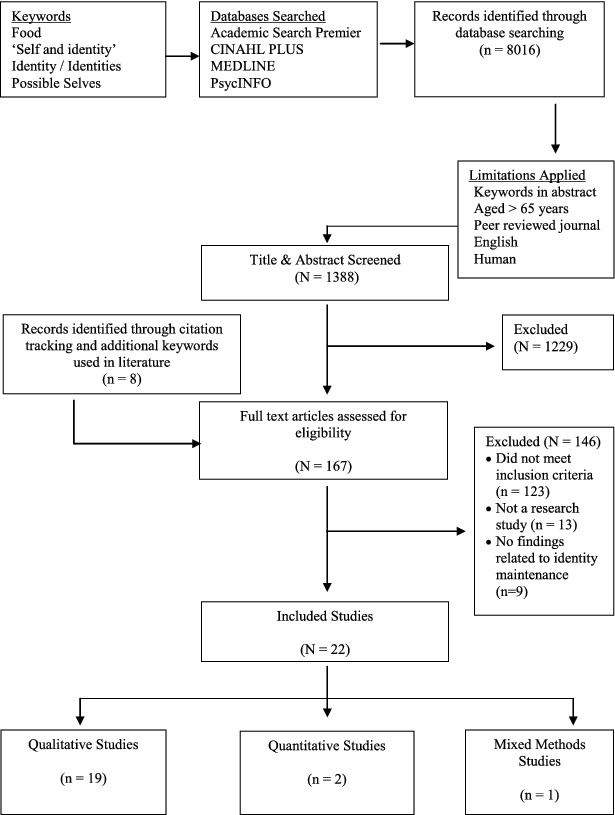
Search strategy results.

The search strategy and results from the four phases of the search strategy are presented in Figure 1. The initial search yielded 8016 abstracts. This was reduced to 1388 papers after limits were applied. Screening of the study titles and abstracts using the inclusion and exclusion criteria reduced the number of papers for review to 159. Eight additional papers were identified in the secondary search. This meant 167 full text articles were reviewed for eligibility using the inclusion and exclusion criteria. Thirty-one studies met the inclusion criteria for the review.

Nine of the thirty-one studies were subsequently excluded, because there was no evidence of maintenance or change in food identities in the full text. Five of the nine excluded studies used quantitative methods. Four of these studies measured the relationship between identity and consumer behaviour (Cook, Kerr, & Moore, 2002; Michaelidou & Hassan, 2008; Schryver & Smith, 2002; Schryver, Smith, & Wall, 2007). One quantitative study measured the relationship between possible selves and health behaviour (Hooker & Kaus, 1992). The remaining four of the nine excluded studies used qualitative methods. One qualitative study explored the uptake of health promotion interventions in relation to health and ageing, using discourse analysis (Pond, Stephens, & Alpass, 2010). Another examined the enactment of ethnic identities among three ethnic groups (Devine et al., 1999). Two other qualitative studies were part of a multinational research consortium investigating meal preparation among women in New Zealand, Thailand, Canada, and the USA (Shordike & Pierce, 2005; Wright-St Clair et al., 2013).

### Maintaining identities through the social aspect to food activities

There was moderate evidence from nine qualitative and two quantitative studies that there is a relationship between participation in food activities and maintenance of social identities in adulthood and later life. The quality of these studies ranged from a low score of 3 (Cantarero, Espeitx, Lacruz, & Martín, 2013) to a high quality score of 18 (O’Sullivan, Hocking, & Wright-St. Clair, 2008), with a median of 12/18. The quality scores of individual studies are presented in Tables 3 and 4. Studies were conducted in a broad range of cultural contexts with both dominant and minority groups in the United Kingdom, United States of America, Canada, New Zealand, Thailand, Spain, and Chile. The narrative analysis found a variety of identities were maintained by cooking traditional meals, giving and receiving love, eating traditional foods, and by shopping.

**Table 3.** The relationship between food activities and identity maintenance–qualitative evidence.

Authors	Research design/data collection	Sampling	Theoretical framework	Data analysis	Evidence of reflexivity	Validation of findings	Score
Maintenance of women's gendered ethnic identities							
Beoku-Betts (1995)	Ethnographic: Semi-structured interviews	*N* = 22 Women Age: 35–75 USA (Gullah)	Black feminist studies Afro-centric values system	Qualitative inductive narrative analysis	Meta-position as Black feminist scholar made explicit	Triangulation of data sources	15
	Journal/field observations						
Hocking, Wright-St. Clair, and Bunrayong (2002)	Qualitative: Focus groups	*N* = 49 Women Age = >60–65 Thai (*n* = 33) New Zealand (*n* = 16)	No	Interpretive analysis narrative approach	No	Triangulation of researchers (data analysis)	12
Wright-St Clair et al. (2005)	Qualitative: Focus groups	*N* = 16 Women Age > 65 New Zealand	No	Interpretive analysis	No	Triangulation of researchers (data analysis)	12
O’Sullivan et al. (2008)	Qualitative: Focus groups	*N* = 20 Women Age: 65–93 Canada	Symbolic interactionism	Interpretive analysis narrative approach	Yes	Member checking Peer review Field notes Immersion Participant observation	18
D’Sylva and Beagan (2011)	Qualitative: In-depth interviews	*N* = 13 Women Age: 26–70 Canada (Goan)	NR	Inductive qualitative analysis	Explicit position of both researchers in relation to study	Theoretical saturation Field notes	15
Janowski (2012)	Food-centred life history methodology: Lightly structured interviews	*N* = 7 Women Age: Born 1913–1938 UK (Polish)	NR	NR	Explicit position of researchers in relation to study	NR	9
Maintenance of ethnic identities							
Hadjiyanni and Helle (2009)	In-depth interviews	*N* = 13 Age: 36–68 Gender: NR USA (Ojibwe)	NR	NR	Design perspective	Research/field observations House plans Photographs	9
Maintenance of community identities							
Griffith (2003)	Semi-structured interviews	*N* = 25 Age: 50–91 Gender: Women (*n* = 20) Men (*n* = 5) USA	Symbolic interactionism	Comparative pattern analysis	NR	No	12
Scarpello et al. (2009)	Semi-structured interviews	*N* = 29 interviews Age: 18–65+ Gender: Women (*n* = 22) Men (*n* = 7) UK	No	Interpretative phenomenological approach	No	Theoretical Saturation Triangulation of researchers	9

**Table 4.** The relationship between food activities and identity maintenance–quantitative evidence.

Authors	Research design	Reliability and validity of measures	Sample size and representativeness	Response rate	Appropriateness of statistical analysis	Control of potential confounders	Score
Cantarero et al. (2013)	Mixed methods. Quantitative phase included cross-sectional participant sample	NR	*N* = 816 Simple random sampling	NR (0)	Inappropriate tests used for type of data	NR	3
Schnettler et al. (2012)	Cross sectional	Measures pilot tested and reliability reported	*N* = 400 (1)	NR	Appropriate and hypotheses tested	Included in analysis	11

Women maintained their gendered ethnic identities by cooking traditional meals. Having a ‘rice culture’ is a central component of a Gullah identity (Beoku-Betts, 1995). In her ethnographic study, Beoku-Betts (1995) found rice was essential for a ‘full meal’ and continuing traditions – such as the strict rituals for preparing rice. A Gullah identity was also maintained in the everyday practices of skilful selection and combination of food seasonings; preparing food from raw ingredients; finding ways to compensate for time pressure; and always cooking a little more for unexpected visitors.

The New Zealander women in Wright-St Clair et al.'s (2005) focus group study with 16 women similarly maintained their identities by preparing and adapting traditional foods, creating new dishes, making everyone feel welcome, and using inherited dishes when cooking for Christmas. Participants validated their individual identities as knowledgeable and skilled cooks by sharing family recipes in the focus group, and by serving and gifting favourite foods to family members at Christmas time. In a focus group study with Canadian women, O’Sullivan et al. (2008) found that the national identities based on the family's cultural heritage were maintained by cooking and serving foods from the family's country of origin, and incorporating foods associated with other cultural heritages when other adults married into the family.

In D’Sylva and Beagan's (2011) exploratory qualitative research with 13 first-generation Goan–Canadian women, Goan identities were maintained through the continued preparation of Goan foods on an almost daily basis, and especially during family celebrations such as Christmas, and at community events. Becoming older and having fewer childcare responsibilities made more time available for traditional cooking, contributing to identity maintenance. D’Sylva and Beagan (2011) also emphasise the importance of foodwork as a source of empowerment within the family and the wider Goan community, because of the value placed on Goan culinary skills.

The desire to cook authentic Polish food is evident in Janowski's (2012) ethnographic study of the role of food and foodwork in identity. Janowski (2012) focused her study on seven Polish women who were deported to Russia during World War II. All were living in one community in the United Kingdom in late 2008 and 2009. ‘Authentic’ food requires the correct ingredients. Across D’Sylva and Beagan (2011), Kohinor, Stronks, Nicolaou, and Haafkens (2011), Hadjiyanni and Helle (2009), and Janowski's (2012) studies, participants experienced difficulty getting the right ingredients as a threat to their identities. ‘Authentic’ food also requires knowledge passed across generations. D’Sylva and Beagan (2011) highlight passing on food knowledge to younger generations as a positive experience. As does Beoku-Betts (1995). In contrast, many of Janowski's (2012) participants were too young to learn from their mothers before deportation. Even as older women, they questioned their ‘Polishness’, because they had not learned to cook Polish food in Poland.

Giving and receiving love through food preparation also maintained women's family identities. In Wright-St Clair et al.'s (2005) study, Christmas meal activities were used to make and remake family identities over time as the family changes through births, marriages, and deaths. Similarly, O’Sullivan et al. (2008) found using meaningful objects handed across generations, and maintaining traditions unique to the family maintained a sense of being a family, within their theme ‘food as love’. In D’Sylva and Beagan's (2011) study, maintenance of a Goan family identity also occurred through the preparation of meals as a way of caring for the family.

In other three studies that included men and women, eating traditional food contributed to the maintenance of ethnic identities. In their quantitative study of life satisfaction and satisfaction with food-related life among the Mapuche in Southern Chile, Schnettler et al. (2012) found participants who consumed Mapuche food only occasionally had a lower life satisfaction (β = −0.406, *p* < 0.1) than those who consumed Mapuche food generally. The authors argue that the life satisfaction and quality of life are explained in part by the maintenance of ethnic identities through food activities. In a mixed-methods study of the relationship between food preferences and cultural identity, Cantatero et al. (2013) found adults aged 55 to 64 and over 65, and those participants who were retired, rated their preference for Aragonese food higher than other participants. Higher preference was also associated with higher consumption of Aragonese products.

In their qualitative study, Hadjiyanni and Helle (2009) found traditional foods are a means to re/claim the past and construct an ethnic or cultural identity for the Ojibwe, by eating traditional foods at least weekly, and a traditional food pattern of eating when hungry. These themes are also evident in D’Sylva and Beagan's (2011) study with Goan–Canadians, and in Kohinor et al.'s (2011) study with Surinamese in the Netherlands. Nevertheless, challenges to identity re/clamation were difficulty in getting traditional foods, inadequate kitchen facilities, and eating boxed convenience foods (Hadjiyanni & Helle, 2009).

There was also limited evidence from two studies that shopping may be important in the construction and maintenance of relational identities across the life course and into later life. In semi-structured interviews with 25 older adults in the United States, Griffith (2003) found shopping as a rite of passage provided an opportunity for the construction of identity, particularly in the early years. In later life, shopping contributed to identity maintenance through the construction of a network of social support in the retail environment, characterised by friendships developed with employees and fellow customers, particularly later in life or after widowhood. Scarpello, Poland, Lambert, and Wakeman (2009) found shopping at the village store was important for the maintenance of a community identity, within the main theme of ‘Village store as icon’.

### Changing food choices, changing health, and changing identities

There was also moderate evidence from nine qualitative studies that changes in the meaning and performance of food activities threatened identity. The studies varied in their quality, with two studies being assessed as weak (Locher et al., 2010; Moss, Moss, Kilbride, & Rubinstein, 2007), and two as very high quality (Atta-Konadu, Keller, & Daly, 2011; Bisogni, Connors, Devine, & Sobal, 2002). Scores ranged between 3 and 18, with a median of 13/18. A summary of the quality assessment for each study is presented in Tables 5 and 6. The finding that changes in meaning and performance of food activities threatened identities was also consistent across a range of contexts. Studies were conducted in the USA, Australia, and Canada. Four studies were conducted in the United Kingdom.

**Table 5.** Qualitative evidence of a threat to the relationship between food Activities and identity maintenance.

Authors	Research design/data collection	Sampling	Theoretical framework	Data analysis	Evidence of reflexivity	Validation of findings	Score
Changes in food choice as a threat to food activities and identity							
Bisogni et al. (2002)	Grounded theory: Focus groups In-depth interviews Theoretical samplint	*N* = 17 Age: 25–89 Gender: Women (*n* = 9), Men (*n* = 8) USA (White)	Constructionist	Constant comparison	Yes	Theoretical saturation	18
Diabetes as a threat to food activities and identity							
Broom and Whittaker (2004)	Discourse and Narrative Analysis	*N* = 119 Age: 20–90 Gender: Women (*n* = 60) Men (*n* = 59) Australia	NR	Thematic analysis – poorly described	No	Diabetes support group Focus group with general practitioners	9
Peel et al. (2005)	Qualitative: Longitudinal over six months	*N* = 40 Age: 21–77 Gender: Women (*n* = 19) Men (*n* = 21) Scottish (White)	Discursive Health Psychology	Thematic discourse analysis	NR	No	12
Kohinor et al. (2011)	Qualitative Semi-structured interviews	*N* = 32 Age: 36 to 70 years Gender: Women (*n* = 20) Men (*n* = 12) Netherlands (Surinamese)	Grounded theory principles (no evidence of application)	Coding matrix	No	Triangulation of researchers	12
Mathew et al. (2012)	Qualitative: Secondary analysis focus groups (*n* = 5) and Individual interviews (*n* = 9)	*N* = 35 Mean age = 57 years Gender: Women (51.4) Men (48.6%) Canada	No	Thematic analysis	Authors had variety of expertise – reduced risk of bias in analysis	Independent coding by three authors	15
Coeliac disease as a threat to food activities and identity							
Rose and Howard (2014)	Grounded theory: Written narratives in a survey	*N* = 130 Age: 19–78 Gender: Women (67%), Men (33%) UK	Narrative psychology No clear theoretical approach to identity	Grounded theory methods (Charmaz, 2006) Described in detail	No	Theoretical saturation Triangulation of researchers	15
Cancer as a threat to food activities and identity							
Locher et al. (2010)	Grounded theory: Semi-structured in-depth interviews	*N* = 30 patients *N* = 21 carers Patient age = 68–90 Gender: Women (*n* = 17) Men (*n* = 13) USA	Constructivist Not evident in analysis	Claim Glaser and Strauss, but not evident in results	No	Theoretical saturation – unlikely given participant sample	3
Valentine (1999)	Qualitative: Case studies	*N* = 7 Age: NR Gender: NR Nationality: British (Yorkshire)	Somers (1994) conceptualisation of identity in narrative	Data analysis methods not clear	No	Substantial detail of each case study	12
Frailty as a threat to food activities and identity							
Moss et al. (2007)	Qualitative: Ethnographic Interviews	*N* = 15 Age: > 75 Gender: Men USA	No	Draw on many methods of qualitative data analysis. Do not describe own methods in detail	Theoretical position of the authors in relation to food literature is made explicit	Coding by one author Discussion of analysis in weekly meetings. No data triangulation	6
Dementia as a threat to food activities and identity							
Atta-Konadu et al. (2011)	Grounded theory: Longitudinal over three years	*N* = 9 dyads Age: 58–88 (T1) Wives with dementia and their husbands Canadian (white)	Grounded theory Symbolic interactionism Role theory	Constant comparison	Theoretical position of the authors in relation to food literature is made explicit	Coding by one author but discussion of analysis in weekly meetings. Longitudinal design	18

**Table 6.** Quantitative evidence of a threat to the relationship between food activities and identity maintenance.

Authors	Research design	Reliability and validity of measures	Sample size and representativeness	Response rate	Statistical analysis	Control of potential confounders	Score
Bradbury et al. (2008)	Cross sectional	Measures verified using Chronbach alpha	Representative but power calculation not reported	131/153 (85%)	Appropriate and hypotheses tested	Included in analysis	14

Bisogni et al. (2002) found there were a variety of reasons why most of the 17 participants in their study had experienced some change in their food identities as a consequence of change in food choice. Health was the primary focus in nine other studies. Studies conducted with participants with diabetes (Broom & Whittaker, 2004; Mathew, Gucciardi, De Melo, & Barata, 2012; Peel, Parry, Douglas, & Lawton, 2005), coeliac disease (Rose & Howard, 2014), cancer (Locher et al., 2010; Valentine, 1999), dementia (Atta-Konadu et al., 2011), and frailty (Moss et al., 2007) consistently showed changes in health led to changes in food activity participation, that in turn threatened or changed identities. In three of the eight studies, a change in health also challenged and/or changed carers’ identities (Atta-Konadu et al., 2011; Locher et al., 2010; Valentine, 1999). The primary reasons why a change in food activities threatened or changed identities were a loss of control, a change in the social aspect to food activities, and changing roles and responsibilities.

The loss of control over food activities was the first important threat to identity. In their narrative study with Australian adults with diabetes, Broom and Whittaker (2004) identity was challenged by blame – for not taking better care of one's health, and for lacking control. Control included diet and food choice, eating fats and sweets, and self-control. In Peel et al.'s (2005) study using a discursive approach, Scottish men and women with type 2 diabetes tried to accomplish and maintain a positive identity as a ‘compliant’ or ‘good’ diabetic by justifying lapses and ‘cheating’. ‘Cheating’ was blamed on particular contexts, like eating out. Mathew et al. (2012) found women were open about their identity as a diabetic, but experienced difficulty with control. Women also used emotionally laden language such as ‘cheating’ to describe their lack of control, in a similar way to participants in Peel et al.'s (2005) study.

It is evident in the text of Locher et al.'s (2010) findings that older women with cancer experience are a threat to their identity as wives, mothers, and a person who is in control. Being unable to prepare meals disrupted these women's ability to carry out their gendered roles within their marriage, and their caring roles with their daughters. The women with cancer in Locher et al.'s (2010) study complained that their husbands did not belong in the kitchen, that the meals prepared were not what they would choose to eat, or that meals they did like were cooked in the wrong way. Locher et al. (2010) ascribe this frustration to a loss of control.

A change in the social aspect to food activities was a second threat to identities. The central theme of Rose and Howard's (2014) grounded theory study of experiences of living with coeliac disease was ‘A changed identity’. Identities were changed through experiences of social invisibility and living with widespread ignorance. Participants experienced exclusion at social events, especially when they were unable to eat the same foods as others or had to be specially catered-for. In Peel et al.'s (2005) study men had difficulty with a diabetic identity and often hid it from family and friends. This meant they either avoided social situations in which their diet would be disrupted, or they lost control and ate the foods on offer. This may be explained by Peel et al.'s (2005) finding that men did not take sole responsibility for their diabetes management, but rather distributed this responsibility to others in the family.

Changing roles and responsibilities was a third threat to identities because of health. In Valentine's (1999) narrative study of the relationship between identity and ‘the home’, Walter's wife's illness and death involved changes in his identity from ‘traditional man’ to ‘new man’. Part of the change in his identity came from the need to learn to cook. For the 15 frail older men, aged over 75 years in Moss et al.'s (2007) qualitative ethnographic study declining health also threatened a masculine identity, because of a loss of ability to perform or do food activities.

Atta-Konadu et al. (2011) investigated the food-related role shifts experienced by Canadian women with dementia and their husbands. The authors describe how men try to maintain their wives’ standards, provide healthy and nutritious meals, and watch over their wives as a way of respecting and maintaining their wives’ role identities. Identities were threatened most in the initial phase of tentative change. Also, this threat had a greater impact on psychological well-being for wives who viewed food roles as ingrained in a feminine identity. For these women, losing responsibility for food activities meant failing to meet gendered expectations, and loss of part of their identity as wives and women. The men in these relationships also experienced a threat to their masculine identities, because they viewed food activities as tedious and not masculine.

The only study in which a change in health did not lead to a change in identity was Bradbury et al.'s (2008) quantitative study of dentate vs. non-dentate adults and older adults. However, Bradbury et al.'s (2008) quantitative study measured health identities, while the other qualitative studies considered a broader range of identities including personal identities (Broom & Whittaker, 2004; Rose & Howard, 2014) and gender identities (Locher et al., 2010; Mathew et al., 2012; Moss et al., 2007; Peel et al., 2005; Valentine, 1999).

## Discussion

Our review is the first meta-analysis to clearly demonstrate a relationship between food activities and the maintenance of identities in adulthood and life. The main focus in the studies identified in the review was the maintenance of women's identities. This is perhaps unsurprising because the assumption that women are primarily responsible for meal preparation has been supported by the literature for some time (Beardsworth & Keil, 1996; Charles & Kerr, 1986; Charles & Kerr, 1988; Dobson, Beardsworth, Keil, & Walker, 1994; Warde & Hetherington, 1994). Even with the advent of the ‘new age man’, women spend more time and take more responsibility for meal preparation (D’Sylva & Beagan, 2011; Pettinger, Holdsworth, & Gerber, 2006; Warde, Cheng, Olsen, & Southerton, 2007). However, it is not clear that food activities maintain a composite sense of self, in addition to component identities for these women.

Studies focusing on other activity domains have also shown a relationship between meaningful activities and identity maintenance. In their qualitative study with three cancer survivors (two women and one man), Reynolds and Prior (2006) found that visual arts were important in the maintenance of participants’ individual and social identities. They also highlight the importance of meaningful leisure activities in the reconstruction of identities following an illness. Similarly, Taylor and Kay (2013) highlight the importance of serious leisure activities as a means to maintain identity among healthy adults. Others have highlighted the importance of driving in the maintenance of identities in later life (Classen, Winter, & Lopez, 2009; Vrkljan & Polgar, 2007). This suggests that older adults use a variety of daily activities to maintain their identities. However, this does not provide conclusive evidence that meaningful activities contribute to the maintenance of a composite sense of self, as suggested by Christiansen (1999).

This study also found a relationship between food activities and a change in identities. Although there are many life experiences which may challenge a positive view of the self (Brandtstadter & Greve, 1994; Kroger, 2007; Sneed & Whitbourne, 2005), our review only found evidence of a change in food activity participation and identities because of changes in food choice and deteriorating health. It was interesting to find that changes in health threatened the social context of food activities, both in the way participants shared meals with others, and shared roles and responsibilities in the household for food activities.

This suggests that changes in the social context of food activities may have important implications for maintenance and change in food identities. However, this review found there is a lack of studies considering other life experiences that can change the social context of food activities. In a study of the impact of widowhood on weight and dietary behaviour in 58 recently widowed men and women with 58 matched married participants, Shahar, Schultz, Shahar, and Wing (2001) found that widowhood led to a number of changes in food activities, including the number of meals widowed men and women eat alone. In a study of the relationship between loneliness and nutritional status, Ferry, Sidobre, Lambertin, and Barberger-Gateau (2005) found 42.6% of participants were not meeting their daily nutritional needs, while 21.3% showed evidence of malnutrition (*N* = 150). Furthermore, in their qualitative study of food-related health perceptions and food habits of 18 Swedish women, aged 65 to 88, Gustafsson and Sidenvall (2002) found meals eaten with others were pleasurable, while women living alone viewed food as a necessity. Similarly, in another study of 18 older men, aged 64 to 84 years, with somatic diseases in Sweden, Kullberg, Björklund, Sidenvall, and Åberg (2011) found only single-living men who had previously been living in a partnership described cooking as a need, instead of pleasure. These four studies do not examine the effect of these changes in food activities on participants’ identities. However, the many changes in food activities experienced by their participants adds weight to our hypothesis that a change in the social context of food activities leads to changes in food activities, and subsequently identities. Aside from the effect of changes in social context on the relationship between food activities and identity maintenance, there are other important gaps in the literature. The inter-relationship between gender, food activities, and identity maintenance remains poorly understood. First, there is more emphasis on men's threatened masculine identities. This means it is unclear if and how men's identities are maintained through participation in food activities. Second, there is an emphasis on the maintenance of women's identities. This means that the relationship between food activities and a change in women's identities has also not been explored in any depth. The unique experience of identity maintenance and change in later life has also not been explored in depth, because only six of the studies included only older adults in the participant sample. Finally, there is no evidence of a relationship between food activities and identity maintenance and change for future-orientated aspect of identity, including possible selves.

Although self, self-concept, and identity are complex phenomena that require multiple research perspectives (Oyserman, Elmore, & Smith, 2012), most of the studies in this review were qualitative studies. At the same time, an exploratory approach to maintenance and change in food identities for urban community-living British older adults is needed because so little food-identity research has been conducted with this group. There is an opportunity to explore the relationship between food activities and maintenance and change in food identities using mixed methods. Mixed-methods research can include the mixing of qualitative methods only (Annells, 2006), quantitative methods only (Creswell, Plano Clark, Gutmann, & Hanson, 2003; Haig, 2005), or a combination of qualitative and quantitative approaches (Creswell et al., 2003; Morse, 2003; Tashakkori & Teddlie, 1998). The benefit of this research design over previous studies is that mixed methods would provide a more comprehensive understanding of the relationship between food activities and identity maintenance, and change than could be achieved with a single method.

### Implications of the review

There is a moderate level of evidence that food activities play a role in the maintenance and change in many different identities. This finding suggests that older adults may be able to maintain their important identities, and a composite sense of self, by participating in food activities. Nevertheless, this review has shown a lack of understanding of the specific challenges older adults face in the maintenance of their food identities. More studies are needed that focus on the experiences of men. There are also substantial gaps in our understanding of what life experiences in later life may challenge the maintenance of identities or lead to changes in identity.

## Conclusion

There is a need to address these gaps in knowledge, so that the role of food activities in the maintenance of psychological well-being, as a component of active ageing, is better understood. This would enable health care professionals to better meet the psychological needs of older adults during nutritional interventions, together with their biological needs.
